# Efficacy of a Mobile Health App (eMOTIVA) Regarding Compliance With Cardiac Rehabilitation Guidelines in Patients With Coronary Artery Disease: Randomized Controlled Clinical Trial

**DOI:** 10.2196/55421

**Published:** 2024-07-25

**Authors:** Celia Cruz-Cobo, María Ángeles Bernal-Jiménez, Germán Calle, Livia Luciana Gheorghe, Alejandro Gutiérrez-Barrios, Dolores Cañadas, Josep A Tur, Rafael Vázquez-García, María José Santi-Cano

**Affiliations:** 1 Faculty of Nursing and Physiotherapy University of Cádiz Cádiz Spain; 2 Institute of Biomedical Research and Innovation of Cádiz Cádiz Spain; 3 Research Group on Nutrition, Molecular, Pathophysiological and Social Issues University of Cádiz Cádiz Spain; 4 Cardiology Clinical Unit Puerta del Mar Hospital Cádiz Spain; 5 Research Group on Community Nutrition & Oxidative Stress University of the Balearic Islands Palma de Mallorca Spain

**Keywords:** coronary event, coronary heart disease, eHealth, lifestyle, mHealth, mobile health

## Abstract

**Background:**

Cardiac rehabilitation is fundamental among patients recovering from a coronary event, and mHealth technology may constitute a useful tool that provides guidelines based on scientific evidence in an entertaining, attractive, and user-friendly format.

**Objective:**

This study aimed to compare the efficacy of an mHealth intervention involving the eMOTIVA app and that of usual care regarding compliance with cardiac rehabilitation guidelines in terms of lifestyle, cardiovascular risk factors, and satisfaction among patients with acute coronary syndrome.

**Methods:**

A randomized controlled clinical trial with a parallel group design was conducted. It included 300 patients (mHealth group, 150; control group, 150) who underwent percutaneous coronary intervention for acute coronary syndrome. Both groups underwent evaluations initially (during hospitalization) and after 3 and 6 months (face-to-face consultations). The eMOTIVA app incorporates a virtual classroom providing audio and video information about a healthy lifestyle, a section for self-recording cardiovascular risk factors, and a section for feedback messages and gamification. The primary outcome variables were (1) adherence to the Mediterranean diet and the frequency of consumption of food; (2) physical activity level, sedentary time, and exercise capacity; (3) smoking cessation and nicotine dependence; (4) level of knowledge about cardiovascular risk factors; and (5) app satisfaction and usability.

**Results:**

The study analyzed 287 patients (mHealth group, 145; control group, 142). Most participants were male (207/300, 69.0%), and the mean age was 62.53 (SD 8.65) years. Significant improvements were observed in the mHealth group compared with the control group at 6 months in terms of (1) adherence to the Mediterranean diet (mean 11.92, SD 1.70 vs 8.92, SD 2.66 points; *P*<.001) and frequency of eating foods (red meat [≤1/week]: 141/143, 97.9% vs 96/141, 68.1%; industrial pastries [<2/week]: 129/143, 89.6% vs 80/141, 56.8%; oily fish [≥2/week]: 124/143, 86.1% vs 64/141, 41.4%; vegetables [≥2/day]: 130/143, 90.3% vs 78/141, 55.3%; fruit [≥2/day]: 128/143, 88.9% vs 85/141, 60.2%; all *P*<.001); (2) physical activity (mean 2112.66, SD 1196.67 vs 1372.60, SD 944.62 metabolic equivalents/week; *P*<.001) and sedentary time (mean 8.38, SD 1.88 vs 9.59, SD 2.09 hours; *P*<.001); (3) exercise capacity (distance: mean 473.49, SD 102.28 vs 447.25, SD 93.68 meters; *P*=.04); and (4) level of knowledge (mean 117.85, SD 3.83 vs 111.00, SD 7.11 points; *P*<.001). App satisfaction was high (mean 42.53, SD 6.38 points), and its usability was excellent (mean 95.60, SD 4.03 points).

**Conclusions:**

With the eMOTIVA app, favorable results were obtained in the intervention group in terms of adherence to the Mediterranean diet, frequency of eating certain foods, physical activity, sedentary time, exercise capacity, knowledge level, systolic blood pressure, heart rate, and blood sugar level. Furthermore, participants reported high app satisfaction and rated its usability as excellent. Thus, this innovative tool is very promising.

**Trial Registration:**

ClinicalTrials.gov NCT05247606; https://clinicaltrials.gov/study/NCT05247606

## Introduction

Cardiovascular disease remains the main cause of death worldwide and is responsible for 17.9 million fatalities every year [1]. In Europe, about 4 million deaths occur each year due to cardiovascular diseases. Although significant progress has been made in the diagnosis and treatment of acute coronary syndrome (ACS), nearly half of these deaths are due to ischemic heart disease [2,3]. In Spain, coronary heart disease (mainly acute myocardial infarction [AMI]) remains the leading cause of death, causing 29,068 deaths per year. Thus, reducing the prevalence of ACS is a crucial objective of public health [4,5].

A large amount of evidence has shown that leading a healthy lifestyle and modifying cardiovascular risk factors (CVRFs), such as stopping smoking, consuming a healthy diet, losing weight, achieving a suitable level of physical activity (PA), and adhering to medication, are vital in the prevention of major adverse cardiac and cerebrovascular events and death in people with coronary artery disease (CAD) [6]. However, a third of patients with CAD do not follow advice about eating healthy, doing PA, and stopping smoking [7].

Owing to medical advances, the mean hospital stay of patients after percutaneous coronary intervention (PCI) has decreased greatly in recent years, meaning that less time is available for providing health care education. Health education plays a fundamental role in the process of cardiac rehabilitation (CR) following ACS, as it empowers patients to take control of their health, improve treatment adherence, prevent future cardiovascular events, and enhance their overall quality of life [8,9]. Providing patients with ongoing support after their hospital discharge may be important after ACS. This should include changes in lifestyle, adherence to medication, and psychosocial well-being [10]. Secondary prevention, which focuses on reducing the risk of recurrent cardiovascular events in individuals who have already experienced ACS, plays a crucial role in the comprehensive management and ongoing care of these patients. CR after AMI is of utmost importance for several reasons. It reduces the risk of experiencing another cardiovascular event. Moreover, CR improves cardiovascular health through a structured program of physical exercise, health education, dietary advice, and emotional support designed to improve the quality of life of people who have experienced ACS. These programs help to control blood pressure, reduce stress, and promote healthy lifestyle habits, which contribute to better cardiovascular health. These programs also contribute to functional recovery. After AMI, many people may experience limitations in their physical and functional abilities. CR can help regain muscle strength, endurance, and cardiac function, allowing patients to return to daily activities and work. Psychosocial support is also critical. CR offers emotional and psychological support, which can be instrumental in helping patients cope with anxiety, depression, and stress closely related to coronary heart disease [11]. However, despite its benefits, less than 50% of patients with coronary heart disease who are eligible for a CR program participate in CR after an acute coronary event. This may be due to limited accessibility and availability owing to a lack of facilities and long waiting lists. Patients may also experience logistical and transport barriers that make regular participation in face-to-face CR sessions difficult [12]. The widespread use of information and communication technology via smartphones may make it easier for health care professionals to handle these patients. Mobile health (mHealth) technology can provide evidence-based health care advice in an entertaining, attractive, and user-friendly format, thereby reducing the cost of health care [13]. In some cases, it may be a viable alternative or complementary approach to conventional CR. This modality involves participation in distance rehabilitation programs that encompass essential elements such as remote counseling, social interaction, supervision, and distance education [14].

A recent meta-analysis [15] concluded that mHealth technology has a positive effect on patients who have experienced a coronary event. It analyzed the effectiveness of different kinds of mHealth programs in changing lifestyle, promoting treatment compliance, and controlling modifiable CVRFs. The analysis found improvements in exercise capacity, PA, physical and mental quality of life, and medication adherence. In addition, readmissions for all causes and cardiovascular causes were lower, although no significant improvements were found regarding blood lipids, arterial blood pressure, BMI, and waist circumference (WC). Another meta-analysis analyzed the effects of mHealth interventions on the risk factors of coronary heart disease, showing that they can lead to significant improvements in BMI, WC, blood lipids, diastolic blood pressure (DBP), and levels of depression. However, no improvements were found in systolic blood pressure (SBP) and anxiety [16].

This clinical trial aimed to assess the efficacy of an mHealth intervention based on a mobile phone health (eMOTIVA) app compared with usual care for improving compliance with CR guidelines and evaluate the secondary prevention outcomes in patients who have experienced ACS. The following variables were assessed: improvements in lifestyle (adherence to the Mediterranean diet, frequency of foods consumed, PA, exercise capacity, sedentary time, smoking cessation, and level of knowledge); control of CVRFs, such as BMI, WC, blood pressure, heart rate (HR), total cholesterol (TC), low-density lipoprotein cholesterol (LDL-C), high-density lipoprotein cholesterol (HDL-C), triglycerides, blood sugar, and HbA_1c_; and usability and satisfaction with the app.

## Methods

### Study Design

We conducted a randomized controlled clinical trial with a parallel group design that included 300 patients with CAD who underwent PCI with stent implantation after ACS. The trial was conducted in the Cardiology Service of a public reference hospital in the south of Spain, in which 1500 PCIs are conducted every year.

The trial has been developed and reported in agreement with the CONSORT (Consolidated Standards for Reporting Clinical Trials) checklist (Multimedia Appendix 1) [17]. The trial was registered at ClinicalTrials.gov (NCT05247606). The study protocol has been previously published [18].

### Participants

During hospitalization, patients were considered eligible to participate if they had experienced myocardial infarction or angina and undergone revascularization with stent implantation, were younger than 75 years, had a smartphone or tablet with internet access for the duration of the study, and were able to manage the software. Patients were excluded if they had an expected survival of less than 1 year, had a physical disability, had severe heart failure, had a severe psychiatric illness, had dementia, did not speak Spanish, had a congenital heart disease with a rheumatic etiology, or required triple heart bypass surgery.

A total of 150 patients were included in each arm. This sample size was considered sufficient to detect a mean effect size (Cohen *d*) of 0.5 [19] with regard to adherence to the Mediterranean diet (mean 8.6, SD 2.0 points) [20], adherence to PA (mean 210.2, SD 221.8 metabolic equivalent (MET)-min/week) [21,22], and a 12% decrease in the prevalence of smokers (prevalence of 21% from the prior pilot study), with a 95% confidence level and a statistical power of 80%.

### Recruitment, Randomization, and Blinding

**Figure 1:**
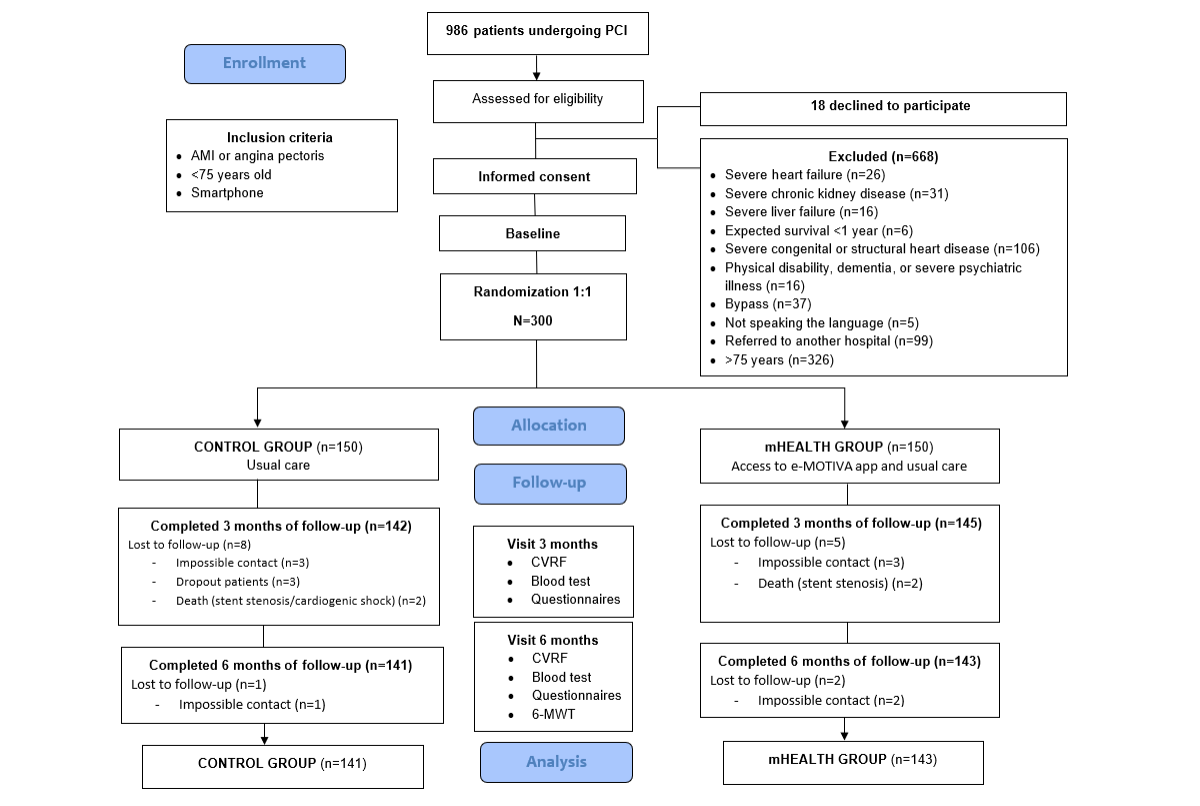
Flow diagram. 6-MWT: 6-minute walk test; AMI: acute myocardial infarction; CVRF: cardiovascular risk factor; PCI: percutaneous coronary intervention.

Recruitment took place between February 2022 and February 2023, and the follow-up continued until September 2023. The flow diagram of participants is shown in Figure 1.

Participants meeting the inclusion criteria described above were randomly allocated using a computerized random number generator (1:1) to either the mHealth or control group (usual care). The researchers analyzing the results were blinded to the allocation of the participants.

### Intervention

**Figure 2:**
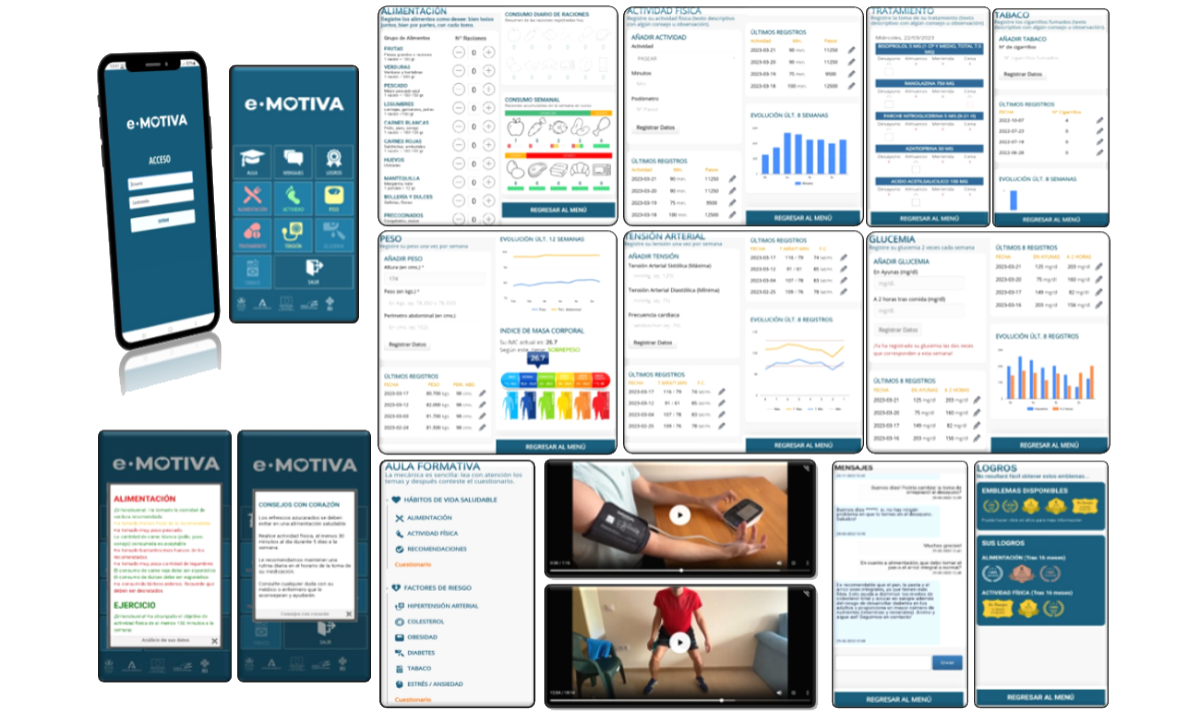
Contents of the eMOTIVA app.

The intervention began while the patient was in the hospital after the coronary event. All participants in the mHealth group had the eMOTIVA app installed on their mobile phones or tablets. The app incorporates a virtual classroom that comprises a space for participation that guides the user using information based on scientific evidence to reach the treatment goals recommended in the clinical practice guidelines and to maintain a healthy lifestyle. This section addresses four cornerstones of the secondary prevention of cardiovascular diseases: (1) healthy lifestyle habits (diet, PA, and recommendations); (2) risk factors (high blood pressure, cholesterol, obesity, diabetes, tobacco use, and stress/anxiety); (3) compliance with the treatment; and (4) goals to be reached regarding diet, PA, body weight, blood pressure, blood sugar, smoking, and treatment. Each section includes online interactive videos (about indoor and outdoor PA, the correct self-measurement of blood pressure and WC, the treatment of cardiac events, and a guided mindfulness relaxation audio). In addition, the classroom provides documents that can be downloaded and printed such as weekly menus and graphics with information (food pyramid, heart health, characteristics and benefits of physical exercise, and recommendations for a healthy lifestyle to stop smoking and control stress). Each section includes a questionnaire that needs to be completed to obtain feedback about the knowledge acquired in the educational sessions. The app includes the use of behavioral strategies to achieve changes in habits through the self-recording of data in the sections related to food consumption, weekly body weight, treatment compliance, PA, smoking, and capillary blood sugar in patients with diabetes. To motivate the participants to improve and maintain healthy habits, the app includes some functions. First, reminders about healthy habits are generated at random on a pop-up screen once a week. Second, personalized messages are provided according to the user’s achievements, and recommendations are adapted to aspects that need to be improved, using information recorded during the previous week. These messages may be green (goal reached), yellow (goal partially reached), or red (goal still to be reached). Furthermore, each icon on the home page of the app appears in the colors according to the goals reached and aspects that need to be improved (Figure 2).

The app uses gamification in the form of achievement icons. Users can obtain different medals if they meet the established recommendations for diet and PA during the months in which they use the app. These systems with fun rewards, such as digital badges obtained for specific objectives, are related to the participation and motivation of users in mHealth interventions, and they encourage an initial and sustained commitment among users to modify CVRFs [23,24]. In addition, gamification can make the intervention more enjoyable, and this is in line with the theory of self-reliance, which assumes that a key part of intrinsic motivation is enjoyment [25]. The app also has fun and colorful warnings and messages, advice, feedback, and self-comparisons through graphics detailing weekly progress (Figure 2).

The app has a messaging section through which the patient can contact health care professionals and resolve any queries (Figure 2). The patients from both groups were evaluated through face-to-face consultations and assessments of medical records at the start and then 3 and 6 months after hospital discharge.

### Outcome Variables

The primary outcome measures at the end of the intervention in both groups were changes in behavior regarding (1) healthy diet (adherence to the Mediterranean diet and frequency of eating each food group); (2) level of PA (METs/week and min/week), sedentary time (hours sitting/week), and exercise capacity (6-minute walk test [6-MWT]); (3) smoking cessation in smokers and nicotine dependence; (4) level of knowledge acquired about CVRFs; and (5) app satisfaction and usability.

The secondary outcome measures were (1) BMI and WC; (2) arterial blood pressure and HR; (3) TC, LDL-C, and triglycerides; and (4) HbA_1c_ and blood sugar in patients with diabetes.

#### Primary Outcomes

Adherence to the Mediterranean diet questionnaire was used (total score: 14 points; <9 points: low adherence, ≥9 points: good adherence) to evaluate diet [20]. The frequency with which each food group was consumed was measured using a food consumption frequency questionnaire (for each food, the participant was required to tick the box indicating the mean frequency of consumption during the last week) [26]. PA time (min/week), intensity (METs/week), and sedentary time (hours seated/week) were analyzed using the International Physical Activity Questionnaire (IPAQ) [27] (light PA: minimum recommended walking of 150 min/week or 495 METs/week, moderate PA: minimum 600 METs/week, and vigorous PA: at least 3000 METs/week). Exercise capacity was measured with the 6-MWT (meters) [28]. Healthy people can walk between 400 and 700 meters in 6 minutes, depending on their age, height, and sex. A greater distance covered is associated with a higher exercise capacity. To this end, a change of 50 meters was established as a clinically significant improvement. A distance below 350 meters is considered a predictor of higher mortality in patients with chronic diseases [29,30]. Smoking cessation was self-reported and nicotine dependence was assessed using the Fagerström test (<4 points: low dependence, 4-7 points: moderate dependence, and 8-10 points: high dependence) [31]. Level of knowledge of CVRFs and a healthy lifestyle were analyzed using a scale validated by the research team in these kinds of patients (maximum score: 120 points). The scale comprises 24 items, each with a score between 1 and 5 points, and respondents were considered to have a high level of knowledge when the correct response was chosen for over 75% of the items (90 points) [32].

#### Secondary Outcomes

During hospital admission and in the physical follow-up visits, the following measurements were taken: body weight and height to calculate BMI, WC, SBP and DBP, HR, lipid values (TC, HDL-C, LDL-C, and triglycerides), HbA_1c_, and blood sugar.

Finally, satisfaction with the app was assessed using a specific questionnaire developed by the research team (maximum score: 50 points; a higher score indicates more satisfaction), and the usability of the app was measured using the System Usability Scale (SUS) questionnaire (total score: 100 points; excellent: >80.3 points, good: 68-80.3 points, poor: 51-67 points, and very poor: <51 points) [33].

### Statistical Analysis

A descriptive statistical analysis was performed. Continuous variables have been summarized as mean (SD), median, SD, 95% CI, and interquartile interval, depending on the distribution of the values (normal or nonnormal), and categorical variables have been summarized as frequency and percentage. At the end of the 6-month follow-up period, the means of the quantitative primary outcomes of the 2 groups (mHealth and control) were compared using the Student *t*-test (variables with normal distribution) and the Mann-Whitney *U* test (variables with nonnormal distribution). The chi-square or Fisher test was used for the comparison of the proportions of qualitative variables between the 2 groups (mHealth and control). A 2-tailed *P*-value of <.05 was considered statistically significant in all tests. SPSS version 24.0 (IBM Corp) was used for the analyses. The researchers analyzing the results were blinded to the allocation of the participants to each group.

### Ethical Considerations

The project was approved by the Costa del Sol Research Ethics Committee, Ministry of Health and Families, Junta de Andalucía and was authorized by the hospital (approval number: 002_jun20_PI-RECAMAR-190. The study complies with Law 14/2007 on Biomedical Research and with the General European Data Protection Regulations, and was conducted following the standards and criteria set out in the latest version of the Helsinki Declaration issued in Fortaleza (Brazil) in October 2013. Moreover, all participants gave their written informed consent to participate in the study.

Concerning the privacy and security of the app, each participant had a private username and password to access the app. Data were stored on a web server and not on a local computer. This web server works with anonymous data and is in Spain to comply with the regulations for the protection of high-level data. The web server performs daily backups of all files, and backups are performed by the software on demand. Thus, the data and program are protected.

## Results

### Overview

During the recruitment period (February 2022 to February 2023), 986 patients underwent PCI and were evaluated for inclusion in the study. Among the patients evaluated, 668 were excluded for not fulfilling the inclusion and exclusion criteria, and 18 refused to participate. In the end, 300 patients were randomized into either the mHealth or control group (150 in each group). There were 9 dropouts in the control group (8 after 3 months and 1 after 6 months). In the mHealth group, there were 7 dropouts (5 after 3 months and 2 after 6 months) (Figure 1).

The baseline characteristics of the participants are shown in Table 1. Most of the participants were male (207/300, 69.0%), and the mean age was 62.53 (SD 8.65) years. In general, both groups were homogeneous.

**Table 1:** Patient baseline characteristics.

Characteristic			Total (N=300)		mHealth^a^ (n=150)		Control (n=150)		*P* value
Male sex, n (%)			207 (69.0)		103 (68.7)		104 (69.3)		.90
**Age (years)**									.005
	Mean (SD)	62.53 (8.65)		61.13 (8.69)		63.93 (8.41)			
	95% CI	61.55-63.51		59.73-62.54		62.57-65.28			
**Educational level, n (%)**									.11
	Primary	128 (44.9)		59 (40.7)		69 (49.3)			
	Middle school	117 (41.1)		60 (41.4)		57 (40.7)			
	High school	40 (14.0)		26 (17.9)		14 (10.0)			
**Employment status, n (%)**									.15
	Employed	94 (32.1)		55 (37.2)		39 (26.9)			
	Unemployed	34 (11.6)		19 (12.8)		15 (10.3)			
	Retired	149 (50.9)		68 (45.9)		81 (55.9)			
	Occupational disability	16 (5.5)		6 (4.1)		10 (6.9)			
**BMI (kg/m^2^)**									.72
	Mean (SD)	28.75 (4.63)		28.84 (4.56)		28.65 (4.70)			
	95% CI	28.22-29.27		28.10-29.58		27.89-29.41			
**Waist circumference (cm)**									.49
	Mean (SD)	103.98 (11.49)		104.44 (11.91)		103.46 (11.02)			
	95% CI	102.59-105.38		102.45-106.43		101.50-105.42			
**Cardiovascular risk factors, n (%)**									
	Overweight	127 (42.3)		64 (42.7)		63 (42.0)		.31	
	Obesity	107 (35.7)		52 (34.6)		55 (36.7)		.31	
	High blood pressure	204 (68.0)		95 (63.3)		109 (72.7)		.08	
	Diabetes	131 (43.7)		58 (38.7)		73 (48.7)		.08	
	Dyslipidemia	199 (66.3)		100 (66.7)		99 (66.0)		.90	
	Smoking	107 (35.7)		56 (37.3)		51 (34.0)		.54	
	Former smoker	116 (38.7)		54 (36.0)		62 (41.3)		.34	
Morbidities^b^			63 (21.1)		25 (16.8)		38 (25.3)		.07
**Personal history of CVD^c^, n (%)**									
	Stable angina	48 (16.0)		21 (14.0)		27 (18.0)		.34	
	Unstable angina	28 (9.3)		10 (6.7)		18 (12.1)		.11	
	NSTEMI^d^	27 (9.0)		8 (5.3)		19 (12.7)		.02	
	STEMI^e^	37 (12.3)		15 (10.0)		22 (14.7)		.21	
	Arrhythmia	17 (5.7)		9 (6.0)		8 (5.3)		.80	
	Stroke	14 (4.7)		9 (6.0)		5 (3.3)		.27	
	Peripheral artery disease	5 (1.7)		3 (2.0)		2 (1.3)		.65	
**LVEF^f^ (%)**									.55^g^
	Mean (SD)	56.79 (10.41)		56.79 (10.10)		56.78 (10.74)			
	95% CI	55.57-58.00		55.12-58.46		55.00-58.56			
**Reason for catheterization, n (%)**									.49
	Stable angina	108 (36.0)		54 (36.0)		54 (36.0)			
	Unstable angina	45 (15.0)		19 (12.7)		26 (17.3)			
	NSTEMI	59 (19.7)		28 (18.7)		31 (20.7)			
	STEMI	88 (29.3)		49 (32.7)		39 (26.0)			
**Stents, n**									.01^h^
	Mean (SD)	2.47 (1.67)		2.26 (1.60)		2.68 (1.70)			
	95% CI	2.28-2.66		2.00-2.52		2.40-2.96			
Complete arterial revascularization, n (%)			244 (81.6)		125 (83.9)		119 (79.3)		.30
**Discharge treatment, n (%)**									
	Anticoagulants	30 (10.0)		18 (12.1)		12 (8.0)		.24	
	Antiplatelets	294 (98.3)		146 (98.0)		148 (98.7)		.64	
	Antihypertensives	289 (96.7)		140 (94.0)		149 (99.3)		.01	
	Insulin	43 (14.4)		14 (9.4)		29 (19.3)		.01	
	Oral antidiabetics	149 (49.8)		66 (44.3)		83 (55.3)		.05	
	Statins	280 (93.6)		141 (94.6)		139 (92.7)		.48	

^a^mHealth: mobile health.

^b^Chronic obstructive pulmonary disease, kidney disease, and obstructive sleep apnea syndrome.

^c^CVD: cardiovascular disease.

^d^NSTEMI: non-ST-segment elevation myocardial infarction.

^e^STEMI: ST-segment elevation acute myocardial infarction.

^f^LVEF: left ventricular ejection fraction.

^g^Mann-Whitney *U* test; median (IQR), mHealth vs control: 58.0 (64.75-52.00) vs 60.0 (65.00-54.00).

^h^Mann-Whitney *U* test; median (IQR), mHealth vs control: 2.00 (3.00-1.00) vs 2.00 (4.00-1.00).

### Outcome Variables

#### Primary Outcomes

The primary outcome variables are shown in Table 2. The score for adherence to the Mediterranean diet was significantly higher in the mHealth group than in the control group after both 3 months (mean 11.63, SD 1.70 points vs mean 9.32, SD 2.55 points; *P*<.001) and 6 months (mean 11.92, SD 1.70 points vs mean 8.92, SD 2.66 points; *P*<.001). The percentage of participants with good adherence to the Mediterranean diet (>9 points) was also significantly higher in the mHealth group than in the control group after 3 months (136/145, 93.8% vs 96/142, 67.6%; *P*<.001) and 6 months (135/143, 94.4% vs 85/141, 60.3%; *P*<.001).

Regarding the frequency of eating food, the consumption of red meat was lower in the mHealth group than in the control group at 3 months (≤1 time/week: 142/145, 97.9% vs 107/142, 75.3%; *P*<.001) and 6 months (≤1 time/week: 141/143, 97.9% vs 96/141, 68.1%; *P*<.001). Moreover, the consumption of industrial pastries was lower in the mHealth group than in the control group at 3 months (<2 times/week: 128/145, 88.2% vs 89/142, 62.7%; *P*<.001) and 6 months (<2 times/week: 129/143, 89.6% vs 80/141, 56.8%; *P*<.001). In addition, the consumption of the following foods was significantly higher in the mHealth group than in the control group: oily fish (≥2 times/week: 116/145, 80.0% vs 68/142, 47.9%; *P*<.001 at 3 months and 124/143, 86.1% vs 64/141, 41.4%; *P*<.001 at 6 months), vegetables (≥2 times/day: 124/145, 85.5% vs 77/142, 54.2%; *P*<.001 at 3 months and 130/143, 90.3% vs 78/141, 55.3%; *P*<.001 at 6 months), fruit (≥2 times/day: 125/145, 86.2% vs 90/142, 63.4%; *P*<.001 at 3 months and 128/143, 88.9% vs 85/141, 60.2%; *P*<.001 at 6 months), and whole-meal cereals (≥1 time/day: 89/145, 61.3% vs 43/142, 30.2%; *P*<.001 at 3 months and 96/143, 66.7% vs 42/141, 29.8%; *P*<.001 at 6 months).

Regarding the time spent doing PA each week (min/week), the mHealth group did significantly more PA than the control group at 3 months (mean 578.10, SD 326.14 min/week vs mean 443.46, SD 278.11 min/week; *P*<.001). Likewise, at 6 months, PA was higher in the mHealth group than in the control group (mean 614.51, SD 332.26 min/week vs mean 408.40, SD 274.49 min/week; *P*<.001). Regarding the intensity of PA (METs/week), the mHealth group performed more intense activity than the control group at 3 months (mean 1991.74, SD 1176.71 METs/week vs mean 1490.48, SD 925.89 METs/week; *P*<.001) and 6 months (mean 2112.66, SD 1196.67 METs/week vs mean 1372.60, SD 944.62 METs/week; *P*<.001). The PA was of moderate intensity in both groups.

The control group had a significantly more sedentary lifestyle than the mHealth group (number of hours seated: mean 9.34, SD 2.13 vs mean 8.57, SD 1.89; *P*=.002 at 3 months and mean 9.59, SD 2.09 vs mean 8.38, SD 1.88; *P*<.001 at 6 months).

Exercise capacity, assessed using the distance covered in meters during the 6-MWT, was significantly higher in the mHealth group than in the control group (mean 473.49, SD 102.28 meters vs mean 447.25, SD 93.68 meters; *P*=.04).

Regarding smoking cessation, although more participants gave up smoking in the mHealth group than in the control group, the difference was not significant. However, the scores for nicotine dependence at 3 months decreased significantly in the mHealth group compared with the control group (mean 2.30, SD 2.27 points vs mean 4.14, SD 2.96 points; *P*=.03).

The level of knowledge of CVRFs and a healthy lifestyle was significantly higher in the mHealth group than in the control group at both 3 months (mean 116.14, SD 4.23 points vs mean 111.02, SD 6.94 points; *P*<.001) and 6 months (mean 117.85, SD 3.83 points vs mean 111.00, SD 7.11 points; *P*<.001).

Finally, the participants in the mHealth group expressed a high level of satisfaction with the app at 3 months (mean 42.32, SD 5.96 points) and 6 months (mean 42.53, SD 6.38 points), and rated it as excellent (>80.3 points) for usability at 3 months (mean 95.75, SD 4.04 points) and 6 months (mean 95.60, SD 4.03 points).

**Table 2:** Primary outcome variables at baseline, and 3 and 6 months.

Variable				Total		Mobile health group		Control group		*P* value	
**Participants, n**											—^a^
	Baseline		300		150		150				
	3 months		287		145		142				
	6 months		284		143		141				
**Mediterranean diet**											
	**Mediterranean diet adherence (score), mean (SD)**										
		Baseline	7.85 (2.52)		7.78 (2.62)		7.92 (2.42)		.48^b^		
		3 months	10.48 (2.45)		11.63 (1.70)		9.32 (2.55)		<.001^b^		
		6 months	10.43 (2.69)		11.92 (1.70)		8.92 (2.66)		<.001^b^		
	**Good adherence, n (%)**										
		Baseline	117 (39.3)		58 (38.7)		60 (40.0)		.57		
		3 months	232 (80.8)		136 (93.8)		96 (67.6)		<.001		
		6 months	200 (77.5)		135 (94.4)		85 (60.3)		<.001		
**Food consumption**											
	**Red meat ≤1/week, n (%)**										
		Baseline	127 (42.5)		63 (42.3)		64 (42.7)		.70		
		3 months	249 (86.7)		142 (97.9)		107 (75.3)		<.001		
		6 months	237 (83.2)		141 (97.9)		96 (68.1)		<.001		
	**Blue fish/oily fish ≥2/week, n (%)**										
		Baseline	120 (40.0)		60 (40.3)		60 (40.0)		.57		
		3 months	184 (64.1)		116 (80.0)		68 (47.9)		<.001		
		6 months	186 (66.0)		124 (86.1)		64 (45.4)		<.001		
	**Vegetables ≥2/day, n (%)**										
		Baseline	98 (32.7)		49 (32.9)		49 (32.6)		.86		
		3 months	201 (70.0)		124 (85.5)		77 (54.2)		<.001		
		6 months	208 (73.0)		130 (90.3)		78 (55.3)		<.001		
	**Fruits ≥2/day, n (%)**										
		Baseline	145 (48.5)		75 (50.4)		70 (46.6)		.30		
		3 months	215 (74.9)		125 (86.2)		90 (63.4)		<.001		
		6 months	213 (74.8)		128 (88.9)		85 (60.2)		<.001		
	**Whole grains ≥1/day, n (%)**										
		Baseline	76 (25.6)		39 (26.4)		37 (24.8)		.76		
		3 months	132 (46.0)		89 (61.3)		43 (30.2)		<.001		
		6 months	138 (48.4)		96 (66.7)		42 (29.8)		<.001		
	**Industrial pastry <2/week, n (%)**										
		Baseline	134 (44.8)		62 (41.7)		72 (47.9)		.55		
		3 months	217 (75.6)		128 (88.2)		89 (62.7)		<.001		
		6 months	209 (73.3)		129 (89.6)		80 (56.8)		<.001		
**Physical activity**											
	**IPAQ^c^ (min/week), mean (SD)**										
		Baseline	387.30 (342.72)		389.81 (355.78)		384.80 (330.33)		.87^b^		
		3 months	511.49 (310.22)		578.10 (326.14)		443.46 (278.11)		<.001^b^		
		6 months	512.18 (321.44)		614.51 (332.26)		408.40 (274.49)		<.001^b^		
	**IPAQ (METs^d^/week), mean (SD)**										
		Baseline	1411.48 (1480.98)		1457.28 (1632.15)		1365.68 (1316.49)		.89^b^		
		3 months	1743.73 (1087.58)		1991.74 (1176.71)		1490.48 (925.89)		<.001^b^		
		6 months	1745.24 (1139.02)		2112.66 (1196.67)		1372.60 (944.62)		<.001^b^		
	**IPAQ H (sitting/week), mean (SD)**										
		Baseline	9.64 (2.40)		9.58 (2.44)		9.69 (2.37)		.84^b^		
		3 months	8.95 (2.04)		8.57 (1.89)		9.34 (2.13)		.002^b^		
		6 months	8.98 (2.07)		8.38 (1.88)		9.59 (2.09)		<.001^b^		
	**6-MWT^e^ (meters), mean (SD)**										
		6 months	460.75 (98.87)		473.49 (102.28)		447.25 (93.68)		.04		
**Tobacco**											
	**Smokers, n (%)**										
		Baseline	107 (35.7)		56 (37.3)		51 (34.0)		.54		
		3 months	42 (42.0)		20 (37.7)		22 (46.8)		.35		
		6 months	42 (43.8)		17 (34.7)		25 (53.2)		.06		
	**Smoking cessation, n (%)**										
		3 months	58 (58.0)		33 (62.3)		25 (53.2)		.35		
		6 months	54 (56.3)		32 (65.3)		22 (46.8)		.06		
	**Nicotine dependence (Fagerström score), mean (SD)**										
		Baseline	5.32 (2.77)		5.39 (2.93)		5.24 (2.62)		.77		
		3 months	3.26 (2.78)		2.30 (2.27)		4.14 (2.96)		.03		
		6 months	3.05 (2.84)		2.18 (2.37)		3.64 (3.02)		.10		
**Cardiovascular risk factors**											
	**CVRF^f^knowledge (score), mean (SD)**										
		Baseline	108.26 (9.34)		108.15 (7.39)		108.37 (10.97)		.40^b^		
		3 months	113.61 (6.27)		116.14 (4.23)		111.02 (6.94)		<.001^b^		
		6 months	114.45 (6.64)		117.85 (3.83)		111.00 (7.11)		<.001^b^		
**App satisfaction (score), mean (SD)**											—
		3 months	—		42.32 (5.96)		—				
		6 months	—		42.53 (6.38)		—				
**App usability (score), mean (SD)**											—
		3 months	—		95.75 (4.04)		—				
		6 months	—		95.60 (4.03)		—				

^a^Not applicable.

^b^Mann-Whitney *U* test.

^c^IPAQ: International Physical Activity Questionnaire.

^d^MET: metabolic equivalent.

^e^6-MWT: 6-minute walk test.

^f^CVRF: cardiovascular risk factor.

#### Secondary Outcomes

The secondary outcome variables are shown in Multimedia Appendix 2. The anthropometric variables (BMI and WC) improved slightly in both groups, with no significant differences between the groups.

SBP was significantly lower in the mHealth group than in the control group at both 3 months (mean 128.96, SD 15.87 mmHg vs mean 133.27, SD 14.85 mmHg; *P*=.01) and 6 months (mean 130.00, SD 21.90 mmHg vs mean 135.78, SD 16.73 mmHg; *P*=.01). However, no significant differences were found in DBP between the groups. HR was significantly lower in the mHealth group than in the control group at 3 months (mean 66.75, SD 8.91 beats/min vs mean 71.93, SD 9.86 beats/min; *P*<.001) but not at 6 months.

The levels of lipid variables (TC, HDL-C, LDL-C, and triglycerides) showed large decreases in both groups, with no significant differences between the groups.

Blood sugar levels were significantly lower in the mHealth group than in the control group at 6 months (mean 101.10, SD 18.57 mg/dL vs mean 115.44, SD 39.46 mg/dL; *P*=.007). However, improvements were not reflected in the HbA_1c_ value.

## Discussion

### Principal Findings

This clinical trial evaluated the efficacy of an mHealth intervention based on the eMOTIVA app with regard to secondary prevention outcomes in patients who experienced ACS. The following variables were assessed: improvements in lifestyle (adherence to the Mediterranean diet, frequency of consumption of foods, PA, exercise capacity, sedentary time, smoking cessation, and level of knowledge) and control of CVRFs (BMI, WC, blood pressure, HR, TC, LDL-C, HDL-C, triglycerides, blood sugar, and HbA_1c_). Our results showed that the eMOTIVA app achieved significantly more favorable results in the intervention group compared with the control group in terms of adherence to the Mediterranean diet, frequency of consumption of foods, time and intensity of PA, sedentary time and exercise capacity, level of knowledge about CVRFs, SBP, HR, and blood sugar. Moreover, the participants reported being very satisfied with the app, and they rated its usability as excellent.

### Primary Outcome Variables

#### Healthy Diet

A healthy diet plays a very important role in both the prevention and treatment of CAD. Strong evidence exists about the efficacy of the Mediterranean diet for managing CVRFs for secondary prevention in patients [34,35]. In our trial, adherence to the Mediterranean diet increased significantly in the mHealth group compared with the control group at both 3 and 6 months. Moreover, in the mHealth group, an increase was observed in the consumption of healthy foods, such as fruits, vegetables, whole-meal cereals, and oily fish, and a decrease was observed in the consumption of red meats and industrial pastries. In a previous study that analyzed a cardiac telerehabilitation program with a mobile care monitoring strategy after ACS, significant improvements were noted in adherence to the Mediterranean diet in the intervention group [36]. By contrast, other authors have not reported significant differences between groups for healthy eating with the use of a support program based on text messages for patients with CAD, type 2 diabetes, or both [37]. Given our results, mHealth technology involving an app may be useful for improving eating behavior and maintaining a healthy diet in these patients compared with interventions based on text messages alone. The clinical benefits of these improvements in diet have been reported. For example, studies have stated that eating fish that is rich in omega-3 polyunsaturated fatty acids, such as oily fish, at least once a week is associated with a 16% decrease in the risk of cardiovascular disease [38]. Likewise, an increase in fiber consumption of 7 g/day is associated with a 9% decrease in the risk of cardiovascular disease [39].

#### Overall PA

PA is a modifiable factor that plays a crucial role in decreasing recurrent coronary events and mortality. The cardiovascular benefits of PA are well known, with recent meta-analyses reporting that it is significantly associated with a decrease in cardiovascular and all-cause mortality in patients with CAD [40-42]. Our results are promising because participants who used the eMOTIVA app performed more PA and were less sedentary. Although PA was self-reported in our trial, an objective test was conducted to measure exercise capacity using the 6-MWT, and participants in the mHealth group were found to have significantly better exercise capacity. Our results are in line with those obtained in other trials in which the effectiveness of mHealth in CAD patients was analyzed [43-46]. Recent meta-analyses have revealed that the use of interactive mobile apps with self-recording and feedback can achieve an increase in the amount of PA performed by participants and an improvement in their functional capacity [15,47].

#### Tobacco Use

Stopping smoking is one of the most effective secondary prevention measures after experiencing ACS [48]. The EUROASPIRE study [49], which assessed smoking cessation rates in patients with CAD in the whole of Europe and had a follow-up of 2-10 years, stated that individuals who stopped smoking showed a reduction in general mortality of nearly 50%. In our study, although no differences were observed between the groups regarding smoking cessation, nicotine dependence after 3 months, measured by the Fagerström test, was significantly lower in the mHealth group than in the control group. A recent meta-analysis [48] that analyzed smoking cessation and risk factors to continue smoking after ACS concluded that the smoking cessation rate after ACS was 45%. These results are similar to our findings, where we observed that 46.8% (22/51) of participants in the control group stopped smoking, while this figure was higher in the mHealth group (32/56, 65.3%), suggesting that our interactive tool helped participants to maintain the willpower to change, possibly owing to the support and motivation they perceived. Another recent meta-analysis [50] found that telehealth interventions had a significant effect on smoking cessation in patients with CAD. By contrast, other meta-analyses did not find significant differences in smoking cessation between groups using telehealth interventions, but these interventions did not use interactive tools with recording, feedback, or gamification [47,51,52].

#### Knowledge of CVRFs

The level of knowledge of CVRFs and a healthy lifestyle in patients is not adequately addressed in trials analyzing the efficacy of mHealth. In our study, the level of knowledge was significantly higher in the mHealth group than in the control group. These results are in line with those obtained by other authors who reported that the use of a social media platform with learning modules significantly increased the knowledge and awareness of CAD [43]. Therefore, interactive and innovative mHealth tools can play a part in increasing the knowledge of a healthy lifestyle. In our study, the virtual classroom incorporated in the app may have been responsible for the observed increase in knowledge.

### Secondary Outcome Variables

#### BMI and WC

A recent meta-analysis that analyzed the efficacy of mHealth for decreasing risk factors related to CAD found significant decreases in both BMI and WC in the intervention group [16]. However, other recent meta-analyses have reported no significant reductions in these anthropometric values with the use of an app [15,47,51,52], which is in agreement with our results. The participants in the mHealth group in our study consumed more vegetables, fruits, whole-meal cereals, and fish, and less red meat and industrial pastries. Moreover, they complied with the recommendation to perform at least 150 minutes of PA per week. Our application, however, was not specifically designed with weight loss in mind, although it did include dietary advice, and losing weight is known to involve more than merely eating healthy food. It is also necessary to limit calorie intake and increase energy expenditure through PA [53].

#### Blood Pressure and HR

In our study, SBP was significantly lower in the mHealth group than in the control group. Our results are in agreement with the results of other studies that analyzed the use of health care apps in patients with CAD [43,54,55]. This clinical benefit is of note because a meta-analysis [56] concluded that a 10-mmHg decrease in SBP reduces the risks of major cardiovascular events by approximately 20%, CAD by 17%, and all-cause mortality by 13%. However, no significant improvements were found in DBP, possibly due to the intensive drug treatment prescribed after a coronary event that had similar effects on patients in both the mHealth and control groups. On the other hand, the significant decrease in SBP found in our study could be explained by greater compliance with antihypertensive treatment among participants using the app or by greater adherence to the Mediterranean diet and an increase in PA. The recent prevention guidelines for cardiovascular diseases state that lifestyle interventions involving a healthy diet and physical exercise among patients with high blood pressure may be enough to control blood readings and even reduce the amount of medication required to control them [57]. Regarding HR, several trials found that mobile technology did not result in significant differences between groups [24,46,58]. By contrast, Dorje et al [43] reported a significant decrease in HR after 6 months through the use of a WeChat platform. In our study, decreases in HR to below 70 beats/min were found after both 3 and 6 months in the intervention group, but only the decrease at 3 months was significant. The higher HR decrease in the mHealth group compared with the control group may be because the mHealth group performed more PA, which has been shown to be related to a decrease in resting HR [59]. Increases in HR have a direct correlation with cardiovascular events. Several kinds of medications, including beta blockers, have been shown to help with the treatment aim of reducing HR in patients with CAD. Thus, an HR below 80 beats/min and close to 70 beats/min is a treatment goal in hypertensive patients with CAD [60].

#### Lipids, HbA1c, and Blood Sugar Values

Keeping blood lipid levels under control is a very important aim in the secondary prevention of cardiovascular diseases [54]. A meta-analysis conducted by Cholesterol Treatment Trialists’ Collaboration [61] reported that the risk of major vascular events decreased by 21% for each 1 mmol/L reduction in LDL-C achieved with statin treatment. In our study, as in other clinical trials on the efficacy of mHealth in patients with coronary disease, blood lipid values decreased drastically, but no significant differences were found between the groups due to the powerful drug treatment received by all patients after a coronary event [37,46,62]. Likewise, a high blood sugar level is also an important risk factor that can lead to the onset and development of CAD. Diabetes mellitus is an important risk factor for AMI and a common comorbidity among patients hospitalized with AMI (present in approximately 30% of cases) [63]. Our study did not find a significant decrease in HbA_1c_. However, blood sugar levels decreased significantly in the mHealth group after 6 months. These findings for HbA_1c_ may also be a result of the intensive drug treatment followed by the patients in both the mHealth and control groups.

#### Satisfaction and Usability

High levels of satisfaction and acceptance of the health care received have been observed to have positive implications for health outcomes and the patient’s experience, thereby reducing health care costs and the use of emergency services [64]. In our study, satisfaction after 6 months of using the app reached a mean of 42.53 (SD 6.38) points out of 50 points, which was considered a high level of satisfaction, while the score for usability reached a mean of 95.60 (SD 4.03) points out of 100 points, which was considered to be excellent. The self-recording of PA, diet, and clinical variables along with positive personalized feedback likely contributed to the high level of satisfaction and usability reported by the participants who used the eMOTIVA app. Other studies that have used mHealth interventions with these patients have also reported high levels of usability of 80.4 points out of 100 [36] and 87.3 points out of 100 [65]. These findings highlight the potential of mHealth apps as useful tools for improving recovery and supporting secondary prevention after a coronary event. They are particularly relevant for populations in which access to a medical center to take part in CR is difficult, either due to living in remote areas or economic reasons.

### Limitations

This study has some limitations. First, one of the inclusion criteria was that patients had to have a smartphone. However, the ever-increasing use of these devices in the lives of people globally suggests that this limitation is of little importance. Due to the nature of the study, as in most trials with digital tools, it was impossible for either the patients or health care staff to be blinded. However, the staff analyzing the data were indeed blinded to the group allocation of each participant. Some variables were self-reported by patients (adherence to the Mediterranean diet and PA), which could have resulted in them overestimating their health-promoting behavior. However, the results were confirmed by other variables that were measured by health care professionals, such as exercise capacity assessed using the 6-MWT and blood pressure. Another possible limitation is that patients in the control group were 2 years older than patients in the mHealth group, and the proportion of patients receiving insulin, oral antidiabetics, and antihypertensives was slightly higher in the control group.

### Strengths

A strength of the study that stands out is the relatively high number of participants included considering that this was a voluntary intervention study using mHealth, and there were very few dropouts. This might imply that the app was easy to use and that the patients were motivated to change their habits. The use of validated questionnaires specific to this population is another strength. In addition, the hospital where the intervention was conducted is a public reference hospital that treats patients from urban and rural areas. Thus, the sample is representative for the generalization of the results. Finally, the educational sessions and app were designed taking into consideration validated psychological theories. Likewise, our eMOTIVA app included setting objectives, self-monitoring of diet and PA, feedback, and gamification, which are resources that have been shown to improve the results obtained with these mHealth tools [36].

### Conclusions

With the use of the eMOTIVA app, favorable results were obtained in the mHealth group compared with the control group in terms of adherence to the Mediterranean diet, frequency of eating certain foods, PA, sedentary time, exercise capacity, level of knowledge of CVRFs, SBP, HR, and blood sugar levels. This trial highlights the potential of mHealth as a complementary or alternative approach to CR programs conducted in medical centers, which are often overburdened. In addition, the participants reported high levels of satisfaction with the app, and it presented excellent usability. Thus, it could be a promising new tool for the CR of patients with CAD in general and for patients who have difficulty attending a health center or hospital in particular.

## Appendix

Multimedia Appendix 1 CONSORT e-HEALTH checklist (V 1.6.1).

Multimedia Appendix 2 Secondary outcome variables at baseline, and 3 and 6 months.

## Abbreviations

6-MWT: 6-minute walk test
ACS: acute coronary syndrome
AMI: acute myocardial infarction
CAD: coronary artery disease
CR: cardiac rehabilitation
CVRF: cardiovascular risk factor
DBP: diastolic blood pressure
HDL-C: high-density lipoprotein cholesterol
HR: heart rate
LDL-C: low-density lipoprotein cholesterol
MET: metabolic equivalent
PA: physical activity
PCI: percutaneous coronary intervention
SBP: systolic blood pressure
TC: total cholesterol
WC: waist circumference
