# Supplementary material for: Efficacy of a Mobile Health App (eMOTIVA) Regarding Compliance With Cardiac Rehabilitation Guidelines in Patients With Coronary Artery Disease: Randomized Controlled Clinical Trial
Source: JMIR Mhealth Uhealth. 2024 Jul 25;12:e55421. doi: 10.2196/55421 (PMC11310647; doi:10.2196/55421)
Supplement: Multimedia Appendix 2 [file mhealth_v12i1e55421_app2.pdf]

Multimedia Appendix 2. Secondary outcome variables at baseline, and 3 and 6 months.

Variable		Total	Mobile health group	Control group	*P* value
**Participants, n**					—[a]
	Baseline	300	150	150	
	3 months	287	145	142	
	6 months	284	143	141	
**BMI (kg/m^2), mean (SD) (95% CI)**					
	Baseline	28.75 (4.63) (28.22-29.27)	28.84 (4.56) (28.10-29.58)	28.65 (4.70) (27.89-29.41)	.72
	3 months	28.29 (4.43) (27.77-28.80)	28.33 (4.48) (27.59-29.07)	28.24 (4.40) (27.51-28.97)	.86
	6 months	28.27 (4.71) (27.72-28.82)	28.22 (4.69) (27.44-28.99)	28.33 (4.74) (27.53-29.12)	.69[b]
**WC[c] (cm), mean (SD) (95% CI)**					
	Baseline	103.98 (11.49) (102.59-105.38)	104.44 (11.91) (102.45-106.43)	103.46 (11.02) (101.50-105.42)	.49
	3 months	102.40 (10.97) (101.06-103.74)	102.56 (11.53) (100.62-104.51)	102.22 (10.35) (100.36-104.08)	.80
	6 months	101.82 (11.36) (100.43-103.21)	101.59 (11.87) (99.58-103.59)	102.09 (10.80) (100.14-104.03)	.72
**SBP[d] (mmHg), mean (SD) (95% CI)**					
	Baseline	132.93 (20.18)	133.53 (19.94)	132.34 (20.47)	.94[b]

		(130.65-135.23)	(130.31-136.75)	(129.04-135.64)	
	3 months	131.11 (15.50) (129.29-132.92)	128.96 (15.87) (126.32-131.59)	133.27 (14.85) (130.80-135.74)	.01
	6 months	132.88 (19.67) (130.57-135.19)	130.00 (21.90) (126.35-133.65)	135.78 (16.73) (132.99-138.58)	.01
**DBP[e] (mmHg), mean (SD) (95% CI)**					
	Baseline	75.41 (11.52) (74.10-76.72)	75.17 (12.53) (73.14-77.19)	75.65 (10.45) (73.96-77.33)	.71
	3 months	74.86 (10.66) (73.61-76.11)	73.68 (9.76) (72.06-75.30)	76.05 (11.42) (74.15-77.95)	.06
	6 months	77.78 (10.92) (76.50-79.07)	76.84 (11.00) (75.00-78.67)	78.74 (10.79) (76.93-80.54)	.14
**HR[f] (beats/min), mean (SD) (95% CI)**					
	Baseline	73.07 (13.94) (71.48-74.65)	72.63 (12.99) (70.53-74.72)	73.51 (14.85) (71.11-75.90)	.70[b]
	3 months	69.33 (9.73) (68.19-70.47)	66.75 (8.91) (65.27-68.23)	71.93 (9.86) (70.29-73.57)	<.001[b]
	6 months	69.31 (10.51) (68.07-70.54)	68.20 (10.13) (66.51-69.89)	70.43 (10.80) (68.62-72.23)	.07[b]
**Glucose (mg/dL), mean (SD) (95% CI)**					
	Baseline	122.27 (51.12) (116.46-128.08)	116.48 (45.55) (109.13-123.83)	128.06 (55.69) (119.07-137.05)	.02[b]
	3 months	111.51 (35.10)	110.58 (35.92)	112.44 (34.38)	.48[b]

		(107.00-116.02)	(104.03-117.13)	(106.15-118.74)	
	6 months	108.10 (31.36) (103.78-112.42)	101.10 (18.57) (97.51-104.70)	115.44 (39.46) (107.61-123.27)	.007[b]
**HbA_{1c} (%), mean (SD) (95% CI)**					
	Baseline	6.32 (1.35) (6.14-6.49)	6.17 (1.32) (5.92-6.42)	6.45 (1.38) (6.20-6.70)	.01[b]
	3 months	6.43 (1.20) (6.06-6.79)	6.61 (1.40) (6.03-7.19)	6.19 (0.84) (5.78-6.60)	.25
	6 months	6.57 (1.01) (6.30-6.84)	6.44 (1.01) (6.04-6.84)	6.69 (1.02) (6.30-7.08)	.36
**TC[g] (mg/dL), mean (SD) (95% CI)**					
	Baseline	168.83 (46.18) (163.52-174.17)	171.97 (47.11) (164.29-179.65)	165.66 (45.16) (158.27-173.05)	.24
	3 months	116.68 (29.83) (112.95-120.82)	117.23 (29.39) (111.71-122.76)	116.54 (30.39) (110.85-122.23)	.86
	6 months	116.34 (30.79) (112.04-120.63)	113.89 (26.35) (104.71-119.06)	118.89 (34.77) (111.92-125.86)	.25
**HDL-C[h] (mg/dL), mean (SD) (95% CI)**					
	Baseline	42.35 (10.95) (40.92-44.33)	42.63 (10.34) (40.92-44.33)	42.08 (11.57) (40.16-43.99)	.67
	3 months	42.35 (12.67) (40.65-44.05)	42.41 (9.67) (40.58-44.25)	42.29 (15.18) (39.38-45.20)	.49[b]
	6 months	44.02 (11.27)	43.80 (11.22)	44.26 (11.38)	.78

		(42.39-45.65)	(41.54-46.07)	(41.87-46.64)	
**LDL-C[i] (mg/dL), mean (SD) (95% CI)**					
	Baseline	101.15 (42.90) (96.18-106.11)	104.39 (43.20) (97.30-111.49)	97.88 (42.48) (90.88-104.87)	.19
	3 months	54.94 (23.15) (51.85-58.04)	54.42 (23.05) (50.04-58.80)	55.47 (23.34) (51.04-59.90)	.79[b]
	6 months	54.63 (24.14) (51.17-58.09)	50.42 (18.67) (46.66-54.19)	59.07 (28.23) (53.22-64.91)	.10[b]
**TG[j] (mg/dL), mean (SD) (95% CI)**					
	Baseline	142.98 (77.80) (134.02-151.94)	141.62 (76.87) (129.09-154.15)	144.36 (78.99) (131.39-157.32)	.96[b]
	3 months	112.61 (56.35) (105.17-120.05)	109.06 (56.86) (98.37-119.76)	116.13 (55.88) (105.66-126.59)	.12[b]
	6 months	107.16 (55.10) (99.45-114.86)	108.81 (61.69) (96.70-120.93)	105.41 (47.47) (95.84-114.98)	.93[b]

[a]Not applicable.

[b]Mann-Whitney *U* test. [Median (IQR)] mHealth vs control: BMI: 6 months [27.30 (31.22-24.97)] vs [27.14 (30.45-24.97)]; SBP: baseline [129.00 (142.00-120.00)] vs [130.00 (146.00-122.00)]; HR: baseline [70.00 (79.75-65.00)] vs [72.00 (83.00-62.00)], 3 months [67.00 (72.00-60.00)] vs [70.00 (80.00-65.00)], 6 months [67.00 (74.50-67.00)] vs [70.00 (80.00-70.00)]; glucose: baseline [103.50 (129.50-90.25)] vs [114.00 (158.50-96.00)], 3 months [93.00 (166.00-88.50)] vs [125.00 (127.00-96.00)], 6 months [90.00 (111.50-83.50)] vs [104.00 (123.00-88.00)]; HbA_{1c}: baseline [5.70 (6.37-5.40)] vs [5.90 (7.15-5.60)]; HDL-C: 3 months [45.50 (51.50-42.25)] vs [41.00 (51.00-33.00)]; LDL-C: 3 months [61.00 (84.50-54.25)] vs [54.00 (91.00-35.00)], 6 months [54.00 (67.50-45.50)] vs [55.00 (77.50-42.75)]; TG: baseline [123.50 (174.75-95.25)] vs

[128.00 (173.00-97.00)], 3 months [130.50 (204.25-100.25)] vs [100.00 (118.00-57.00)], 6 months [100.00 (132.00-78.00)] vs [112.50 (123.00-78.75)].

[c]WC: waist circumference.

[d]SBP: systolic blood pressure.

[e]DBP: diastolic blood pressure.

[f]HR: heart rate.

[g]TC: total cholesterol.

[h]HDL-C: high-density lipoprotein cholesterol.

[i]LDL-C: low-density lipoprotein cholesterol.

[j]TG: triglyceride.
